# Influence of Football Match-Play on Isometric Knee Flexion Strength and Passive Hip Flexion Range of Motion in Football Referees and Assistant Referees

**DOI:** 10.3390/ijerph182211941

**Published:** 2021-11-13

**Authors:** Vicente Fernández-Ruiz, Álvaro López-Samanes, Juan Del Coso, José Pino-Ortega, Javier Sánchez-Sánchez, Pablo Terrón-Manrique, Marco Beato, Víctor Moreno-Pérez

**Affiliations:** 1 Exercise Physiology Group, School of Physiotherapy, Faculty of Health Sciences, Universidad Francisco de Vitoria, 28223 Madrid, Spain; vicente.fernandez@ufv.es (V.F.-R.); p.terron.prof@ufv.es (P.T.-M.); 2 Centre for Sport Studies, Rey Juan Carlos University, Fuenlabrada, 28943 Madrid, Spain; juan.delcoso@urjc.es; 3 Department of Physical Activity and Sport, University of Murcia, 30720 Murcia, Spain; pepepinoortega@gmail.com; 4 Faculty of Sport Sciences, Universidad Europea de Madrid, 28670 Madrid, Spain; javier.sanchez2@universidadeuropea.es; 5 Comité Técnico de Árbitros (CTA) de la Real Federación Española de Fútbol (RFEF), 28230 Las Rozas, Spain; 6 School of Health and Sports Sciences, University of Suffolk, Ipswich IP4 1QJ, UK; m.beato@uos.ac.uk; 7 Sports Research Center, Miguel Hernandez University of Elche, 03202 Alicante, Spain; vmoreno@goumh.umh.es; 8 Center for Translational Research in Physiotherapy, Department of Pathology and Surgery, Miguel Hernandez University of Elche, 03550 Alicante, Spain

**Keywords:** soccer, fatigue, match competition, physical demands, muscle injury

## Abstract

The aim of this study was to examine the acute effect of officiating a football (soccer) match on isometric knee flexion strength and passive hip flexion range-of-motion (ROM) in referees and assistant football referees. Twelve referees (25.3 ± 3.3 years) and twenty-three assistant referees (25.1 ± 4.8 years) underwent measurements on isometric knee flexion strength and passive hip flexion ROM before and after officiating an official football match. Referees’ and assistant referees’ running patterns were monitored during the match using GPS technology. In comparison to pre-match values, referees reduced their isometric knee flexion strength (−12.36%, *p* = 0.046, Effect size [ES] = −0.36) in the non-dominant limb, while no significant differences were reported in the dominant limb (−0.75%, *p* = 0.833, ES = −0.02). No effect of the match was found in hip flexion ROM values in dominant (−4.78%, *p* = 0.102, ES = −0.15) and non-dominant limb (5.54%, *p* = 0.544, ES = 0.19). In assistant referees, the pre-to-post-match changes in isometric knee flexion strength (dominant limb −3.10%, *p* = 0.323, ES = −0.13; non-dominant limb −2.18%, *p* = 0.980, ES= 0.00) and hip flexion ROM (dominant limb 1.90% *p* = −0.816, ES = 0.13; non-dominant limb 3.22% *p* = 0.051, ES = 0.23) did not reach statistical significance. Officiating a match provoked a reduction in isometric knee flexion strength in the non-dominant limb of football referees, while no differences were reported in assistant referees.

## 1. Introduction

Football (soccer) is an explosive team sport where players perform short and repeated periods of high-intensity anaerobic exercise interspersed with recovery periods of lower intensity [1,2]. At the professional level, football players cover more than 10 km per match with ~600 m at above 21 km/h [3,4]. Consequently, professional football referees need an optimal physical condition to ensure that they follow the course of the game during the explosive movements, changes of directions and sprints performed by the players. It is worth to mention that referees have to be in the correct location and at the right time in every match play of the game and they need to follow players’ actions closely, interpret the rules and make critical decisions within seconds. For these reasons, main referees cover during official matches between 10 and 12 km [5,6] where approximately 18.6% of the total distance is performed at high-speed running (i.e., speeds from 18.0 to 24.9 km h^−1^) [5] including a mean of 38 ± 17 sprints at above 25 km·h^−1^ [7] being match running distance approximately half in the case of assistant referees [8]. However, assistant referees need to perform brief but intense bouts of lateral running to keep level with the most forward striker to adequately judge offsides [9]. Both elite referees and assistant referees may officiate up to 40 matches per season, including national and international competitions [10]. The combination of acute and chronic loads in elite football refereeing may lead to fatigue and to a high risk of injury, particularly during congested calendars.

In this regard, previous studies have described an incidence of 2.0 to 19.6 injuries per 1000 h of match officiating [11,12,13], while this considerable injury incidence may be due to the high physical demands needed to follow the game [14]. Additionally, more than half of both main referees and assistant referees report at least one injury per season [12]. Most of these injuries have been reported to occur in the thigh and frequently as hamstring muscle injury [14]. In order to successfully implement preventive measures to reduce the risk of injury in referees, the identification of the risk factors associated with the occurrence of hamstring muscle injury is essential. To date, the information about risk factors associated with hamstring muscle injury in football referees is scarce and, sometimes, conclusions are incorrectly obtained by extrapolating results from football players.

Previous studies conducted in football players and in athletes of other field-based team sports have labeled several non-modifiable factors associated with the risk of hamstring muscle strain injuries. Among others, increased age, the existence of a previous injury in the same location and ethnicity [15,16] are associated with the likelihood of suffering a hamstring muscle injury. Additionally, there are modifiable factors such as muscle flexibility [17], fatigue [18] and muscle strength [15,16,19,20] which can be modulated to reduce the likelihood of hamstring muscle injury. Modifiable risk factors such as muscle weakness [21] and poor hamstring muscle flexibility [22,23] have received much attention in the literature. It is well recognized that approximately 60% of hamstring muscle strains identified in football players were associated with a high-speed running action [24] and it has been speculated that the injury is produced due a failure of the tissues to tolerate the forces applied during the sprint [15]. Furthermore, decreased isometric knee flexion strength increases the risk of re-injury after an acute hamstring injury [25]. Related to hamstring flexibility, previous studies have showed a relationship between reduced hamstring muscle flexibility and an increased risk of hamstring injury in Belgian [23] and English [17] football players.

A previous study demonstrated fluctuations in hamstring muscle strength and flexibility after simulated match-play in football players [26]. These decrements of lower limb function after match-play in football players could lead to suboptimal recovery, inadequate overall preparation and to an overall higher risk of injury [27]. However, the information about risk factors associated to hamstring muscle injury in football referees is scarce and sometimes, conclusions are incorrectly obtained by extrapolating results from football players. Thus, the aim of this study was to examine the acute impact of football refereeing during an official match on isometric knee flexion strength and passive straight leg elevation (i.e., a measurement of passive hip flexion range of motion; [ROM]) in referees and assistant referees.

## 2. Materials and Methods

In this prospective observational study, 12 male football referees (age: 25.3 ± 3.3 years; body weight: 75.5 ± 12.9 kg; height: 1.81 ± 0.05 m; body mass index, 22.8 ± 1.3 kg·m^2^) and 23 male assistant referees (age: 25.0 ± 9.9 years; body mass: 73.0 ± 9.9 kg; height 1.78 ± 0.02 m; body mass index, 23.0 ± 2.4 kg·m^2^) volunteered to participate. Regarding the selection of referees, all active referees in the 2°B category of Spanish football were invited to participate and the participants in this study was configured with the referees that accepted to take part in this investigation. The following criteria were considered as grounds for exclusion from the current investigation: (a) history of pain of any kind or injury during the match; (b) <90 min of refereeing in a match; (c) non completion of regular training during the two prior weeks to the match; (d) prior hamstring injury in the year before the study. All the measurements were carried out between September and October 2019 and referees and assistant referees have already refereed between 3 and 5 football matches in this season phase. Written informed consent was obtained from all participants and the University of Francisco de Vitoria Bioethics Commission (number 45/2018) approved the study, which complied the recommendations of the latest version of the Declaration of Helsinki.

### 2.1. Experimental Approach

The effects of football refereeing during an official match on isometric knee flexion strength and passive straight leg elevation test were evaluated in both lower limbs by two experienced physical therapists. The order of the test and the order of the selection of the limb being tested were randomly chosen prior to the match test as suggested by a previous study [26]. Referees and assistant referees were tested twice: (a) 60 min prior to the onset of the match and (b) immediately after the end of the match. Before the first measurement, all participants underwent a standardized warm-up consisting of 5 min of running at low intensity (10 km·h^−1^) and dynamic warm-up exercises, as previously suggested [28]. A week before testing, referees and assistant referees were familiarized with all testing procedures to reduce the influence of the learning effect on the results of the investigation. During the official match, referees’ and assistant referees’ running patterns were monitored using a portable GPS unit (Wimu ProTM, RealTrack Systems, Almeria, Spain). Additionally, participants’ internal load imposed by the match was calculated by the session rating of perceived exertion [29]. During the whole duration of each match, air temperature and humidity were measured with a portable weather station (WMR 108, Mextech, India; [28]). All matches were completed on an artificial grass pitch (AT; fibre: monofilament of polyethylene of 60 mm in height) within a dimension of ~100 × 70 m.

### 2.2. Procedures

#### 2.2.1. Isometric Knee Flexion Strength

Hamstring strength was measured with a hand-held dynamometer (0–500 N range, 0.2 N sensitivity; Nicholas Manual muscle test, Co., Lafayette, IN, USA), as previously described [30]. For this measurement, the participant laid down on a bench in prone position with 0° degrees hip flexion and the knee at 15° flexion (i.e., measured inclinometer (ISOMED, Portland, OR, USA)) with the feet just hanging over the edge of the bench [31]. The first examiner placed the hand-held dynamometer on the distal portion of the lower limb, 3 centimeters above the bimalleolar line. The second examiner set the participants pelvis over the sacrum, to avoid its elevation or any kind of movement during the test. The examiner carrying the hand-held dynamometer requested the participant to bend his knee, with the purpose of bringing the heel of the foot to his ipsilateral gluteus. During the measurement, participants performed a maximal contraction lasting ~4 s while the examiners assured the correct position of the participant, as explained above. Each limb was tested 2 times, with a 30-s rest between both measurements. Isometric knee flexion strength was recorded for each limb as the maximal knee torque per kilogram of body weight (N·kg^−1^) obtained during the 2 repetitions of the test. The ICC for this test was 0.83 [31].

#### 2.2.2. Straight Leg Elevation Test

Passive hip flexion ROM was tested with the knee extended to perform the Straight Leg Elevation Test, while it was measured with a manual inclinometer (ISOMED inclinometer, Portland, Oregon). The measurement was performed in the dominant and non-dominant limb according to previous recommendations [32]. For this measurement, the inclinometer was placed on the lateral malleolus on the leg under examination and the telescopic arm was applied parallel to an imaginary bisecting line of the leg while participants were laid down in supine position on a bench. On command, participants elevated his leg while maintaining the aforementioned position and the end of the straight leg elevation test was defined as the point at which the examiners noted compensations that could increase the hip flexion ROM. Two trials were recorded on each limb and the mean value for these two measurements was used for further analysis. The ICC for this test was 0.92 [33].

#### 2.2.3. Internal Load during the Match

The subjective internal load was calculated using the rating of perceived exertion (RPE; 1–10-point scale) which was obtained 30 min after the end of the match, as previously suggested [29]. Referees and assistant referees were familiar with the scale before starting the study. The value of RPE obtained at the end of the match was multiplied by the refereeing time in minutes to obtain the session RPE (sRPE) in arbitrary units (AU).

#### 2.2.4. External Load during the Match

External match load was recorded by GPS units (Wimu ProTM, RealTrack Systems, Almería, Spain) which have shown a good level of accuracy to assess running patterns during football [34]. The device was positioned inside a vest, and it remained between the participants’ shoulders without hindering any movement to officiate the match. The GPS devices obtained data with a frequency of 10 Hz. The total distance covered was measured and categorized afterwards in five speed zones: stationary/walking (<7.2 km·h^−1^), jogging (7.2–13.0 km·h^−1^), running (13.1–18.0 km·h^−1^), high-intensity running (>18.0 km·h^−1^) [35]. In addition, maximal speed (km/h), explosive distance (distance with accelerations >2 m·s^−2^, decelerations <−2 m·s^−2^ and high intensity break distance were measured [35].

### 2.3. Statistical Analysis

Data are presented as means and standard deviation for each sample of referees and assistant referees. The Shapiro–Wilk test was used to assess normal distribution of data. Pre-to-post match values of isometric knee flexion strength and passive hip flexion ROM within each group were compared using Student’s *t*-tests for related samples. The external match load was compared between football referees and assistant referees using the Student’s *t*-test for independent samples. Statistical significance was set at *p* < 0.05 and reported to indicate the strength of the evidence alongside the effect size. Cohen’s effect size (ES) was calculated, and its magnitude categorized using the following criteria: trivial (0–0.19), small (0.20–0.49), medium (0.50–0.79) and large (0.80 and greater) [36]. All the statistical analyses were performed using the SPSS software version 20 (SPSS Inc., Chicago, IL, USA).

## 3. Results

### 3.1. Internal and External Match Load

In referees, RPE was 5.07 ± 1.71 points the end of the match and the sRPE was 456.92 ± 153.93 AU. Main referees covered a mean distance of 10,647.08 ± 2370.67 m across the different speed thresholds (Table 1). In assistant referees, RPE was 3.59 ± 1.60 points and sRPE was 322.82 ± 144.00 AU. Assistant referees covered a mean distance of 6030.31 ± 1791.78 m across the different velocity thresholds (Table 1). The total distance covered by referees was higher than the distance covered by assistant referees (*p* = <0.001, ES = 2.20 [1.33, 3.06]. 

### 3.2. Isometric Knee Flexion Strength 

**Figure 1:**
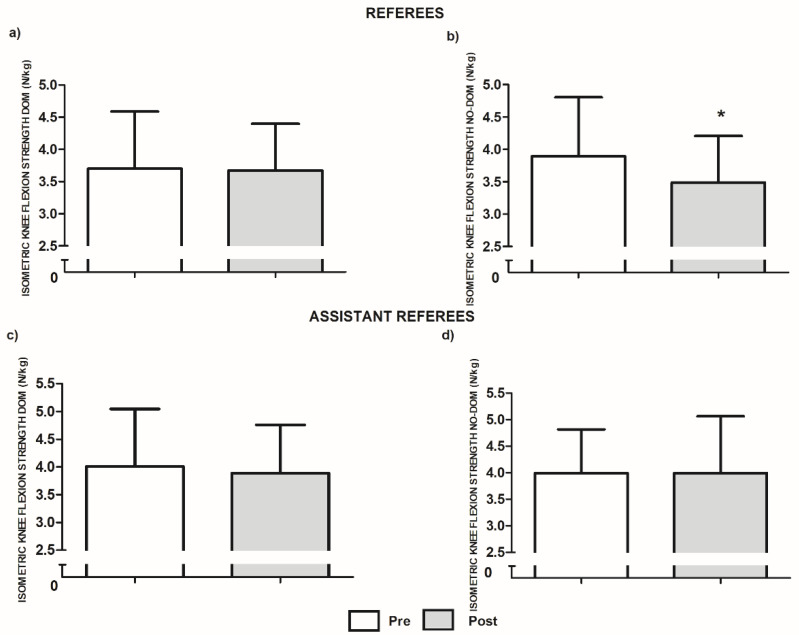
Isometric knee flexion strength values in referees and assistants. N = newton; kg = kilograms; DOM = dominant; NO-DOM = non-dominant. * Significant differences compared to the pre values at *p* ≤ 0.05.

In referees, officiating the match did not induce statistical differences in isometric knee flexion strength in the dominant limb (−0.75%, *p* = 0.833, ES [95% CI] = −0.02 [−0.81, 0.79]) but it reduced this variable in the non-dominant limb (−12.36%, *p* = 0.046, ES [95% CI] = −0.36 [−0.23, −0.49]; Figure 1a,b). In assistant referees, officiating the match did not induce any statistical difference in isometric knee flexion strength in the dominant limb (−3.10%, *p* = 0.323, ES [95% CI] = −0.13 [−0.70, 0.45]) nor in the non-dominant limb (−2.18%, *p* = 0.980, ES [95% CI] = 0.00 [−0.58, 0.58], Figure 1c,d).

### 3.3. Passive Hip Flexion ROM

Officiating the match did not produce differences in the pre-to-match hip flexion ROM in the dominant (−4.78%, *p* = 0.102) and non-dominant limb (5.54%, *p* = 0.544) of referees. Likewise, the match did not induce any change in hip flexion ROM of assistant referees (1.90–3.22%; *p* = 0.816–0.051) for the dominant and non-dominant sides, respectively; Table 2).

## 4. Discussion

The main results of this study showed that officiating a football match produced a decrease in isometric knee flexion strength in the non-dominant limb of main referees, while no other significant differences were found in hamstring muscle strength of the dominant side nor in hip flexion ROM. In assistant referees, the match did not induce any statistically significant change in hamstring muscle strength nor in hip flexion ROM. Overall, these data reflect an acute effect of refereeing on hamstring muscle strength in the non-dominant side of referees.

Muscle strength in the lower limb is essential to produce explosive actions in football (e.g., sprints, accelerations, changes of direction, etc.). However, the repetition of these actions during the match may induce progressive acute fatigue that may reduce both muscle strength and high velocity running performance. In the current experiment, main referees covered ~10.6 km, including ~0.8 km at above 18 km/h^−1^. In addition, main referees covered ~1.2 km while accelerating at >2 m·s^−2^ and reached a value of maximal speed of 27 km/h, in the lower range of professional football players [37]. As a result, isometric knee flexion strength in the non-dominant limb decreased −12.36% at post-match testing compared with pre-match levels, which are in line with previous results obtained in football players [26]. The most likely explanation for this reduction is the appearance of acute fatigue induced by the high physical demands of refereeing. The data gathered in this investigation does not explain why this sign of hamstring muscle fatigue was not present in the dominant limb, but a non-significant reduction of 0.75% was also present in this side. From epidemiological point of view, this finding suggests a higher likelihood of hamstring muscle injury in referees towards the end of the match, as hamstring muscle fatigue and weakness in a potent predictor of hamstring injury occurrence in football players [20,21]. On the other hand, assistant referees covered a lower running distance than main referees at all speed thresholds above 7 km·h^−1^ which may explain that isometric knee flexion values post-match were not different from pre-match measurement in this subgroup of football officials. Differences between referees and assistant referees could be explained due to the lower running demands in the group of assistant referees at high-speed velocities (>18.1 km·h^−1^) [5] because there is a higher activation of hamstrings than when running at lower velocities. Another possible explanation for the lack of hamstring muscle fatigue induced by the match in the group of assistant referees could be associated with the different movement patterns during the match between main referees and assistant referees. This is because a high portion of the distance covered during match by assistant referees is produced with lateral movements, which require a lower involvement of hamstring musculature in comparison to a forward sprint [38]. On the contrary, main referees follow the match play by using forward running in order to maintain visual contact with the ball and the players around it. 

In addition to hamstring muscle fatigue, a reduced hip flexion ROM measured by the Straight Leg Raise test has been linked to a higher risk of hamstring muscle injury [23,39]. However, the current findings indicate lack of significant changes in hip flexion ROM induced by refereeing an official match in both main referees and assistant referees. To the best of knowledge, only one study examined the straight leg raise ROM after match-play in football players and found similar results [26]. As football match-play activity habitually increases ankle ROM after a soccer match-play [40], it can be suggested that an acute football match do not produce significant changes on leg ROM that may entail a higher predisposition to hamstring injury for referees or assistant referees. 

This study has some limitations that should be clearly discussed to understand the true utility of the experiment. First, all the football matches were played in Spanish 2nd B division of the Spanish football league. Although both players and referees are subjected to training programs comparable to professional counterparts, it is probable that the physical demands of football refereeing slightly change with the level of the match officiated [8]. Future investigations should determine the changes in hamstring muscle strength and hip flexion ROM induced in first division and international matches. Second, all the matches were refereed on artificial grass pitches. Recent data indicate that running on artificial turf generates higher peak forces than on a natural grass [41]. Hence, the current outcomes should be compared from that obtained when officiating a match on natural grass. Furthermore, before drawing firm conclusions, future research is needed to verify the reduction in isometric hamstring strength of the non-dominant limb found in this study, although the reduction found in the current investigation is within the margin of error of the measurement (3–12%) [31]. Future investigations should determine the effect of chronic exposure induced by refereeing in a congested calendar or by the accumulation of matches across the season on these potential risk factors for hamstring muscle injury.

## 5. Conclusions

This study showed that officiating a football match provoked a reduction in isometric knee flexion strength in the non-dominant limb in main referees, while no differences were reported in assistant referees. The present data reflect hamstring muscle fatigue during the match which may lead to an increased risk of hamstring muscle injury in football referees. On the other hand, it seems that assistant referees are probably at lower risk of hamstring muscle injury, at least when considering hamstring muscle fatigue as a risk factor. This is probably due to the lower physical demands associated with the role of assistant referees during the match, at least in comparison to main referees. As a practical application, main referees should be involved in strength training programs to reduce the likely decline in hamstring muscle strength induced by a match. Additionally, baseline isometric knee flexion strength should be restored before refereeing the next match.

## Figures and Tables

**Table 1:** Running parameters in referees and assistant referees while officiating an official football match.

Variable (Units)	Referees (*n* = 12)	Assistant Referees (*n* = 23)	*p*-Value	ES [95% IC]
Running distance at 0.0–7.1 km/h (m)	4067.8 ± 1196.8	3456.2 ± 1338.3	0.193	0.48 [–0.21, 1.19]
Running distance at 7.2–13.0 km/h (m)	3530.0 ± 595.7	1649.1 ± 470.9	<0.001 *	3.50 [2.43, 4.58]
Running distance at 13.1–18.0 km/h (m)	2251.6 ± 917.1	718.6 ± 232.9	<0.001 *	2.29 [1.43, 3.15]
Running distance at >18.1 km/h (m)	797.6 ± 417.0	202.1 ± 108.6	<0.001 *	1.95 [1.14, 2.77]
Maximal running speed (km/h)	27.1 ± 2.2	24.9 ± 1.9	0.005 *	1.05 [0.33, 1.77]
Number of accelerations (>2 m/s^−2^)	2926.7 ± 520.2	3360.5 ± 689.8	0.060	0.71 [–0.01, 1.43]
Number of decelerations (<−2 m/s^−2^)	2927.3 ± 520.4	3360.60 ± 690.6	0.060	0.71 [–0.01, 1.43]

* Significant differences comparing referees and assistant referees values at *p* ≤ 0.05.

**Table 2:** Hip flexion range of motion in referees and assistant referees before and after officiating an official football match.

	Referees			Assistant Referees		
Variable (Units)	Pre-Match	Post-Match	ES [95% CI]	Pre-Match	Post-Match	ES [95% CI]
Hip flexion dominant limb (°)	80.42 ± 23.88	76.58 ± 18.09	−0.15 [−0.95, 0.65]	77.74 ± 9.97	79.22 ± 12.77	0.13 [0.45, −0.71]
Hip flexion non-dominant limb (°)	71.66 ± 9.97	75.63 ± 18.14	0.19 [−0.62, 0.99]	77.04 ± 9.98	79.52 ± 11.27	0.23 [−0.35, 0.81]

## Data Availability

Not applicable.
